# Impact of Comorbidities on Beneficial Effect of Lactated Ringers vs. Saline in Sepsis Patients

**DOI:** 10.3389/fmed.2021.758902

**Published:** 2021-12-13

**Authors:** Chien-Hua Tseng, Tzu-Tao Chen, Ming-Cheng Chan, Kuan-Yuan Chen, Sheng-Ming Wu, Ming-Chieh Shih, Yu-Kang Tu

**Affiliations:** ^1^Institute of Epidemiology and Preventive Medicine, National Taiwan University, Taipei, Taiwan; ^2^Division of Pulmonary Medicine, Department of Internal Medicine, School of Medicine, College of Medicine, Taipei Medical University, Taipei, Taiwan; ^3^Division of Critical Care Medicine, Department of Emergency and Critical Care Medicine, Shuang Ho Hospital, Taipei Medical University, Taipei, Taiwan; ^4^Division of Pulmonary Medicine, Department of Internal Medicine, Shuang Ho Hospital, Taipei Medical University, Taipei, Taiwan; ^5^Division of Critical Care and Respiratory Therapy, Department of Internal Medicine, Taichung Veterans General Hospital, Taichung City, Taiwan; ^6^College of Science, Tunghai University, Taichung City, Taiwan; ^7^Graduate Institute of Clinical Medicine, College of Medicine, Taipei Medical University, Taipei, Taiwan; ^8^Department of Dentistry, National Taiwan University Hospital, National Taiwan University, Taipei, Taiwan

**Keywords:** fluid therapy, intensive care, resuscitation, saline, lactated Ringers

## Abstract

**Background:** Lactated Ringers reduced mortality more than saline in sepsis patients but increased mortality more than saline in traumatic brain injury patients.

**Method:** This prospective cohort study was conducted in a medical intensive care unit (ICU) in central Taiwan. We applied standard sepsis evaluation protocol and identified heart, lung, liver, kidney, and endocrine comorbidities. We also evaluated resuscitation response with central venous pressure, central venous oxygen saturation, and serum lactate level simultaneously. Propensity-score matching and Cox regression were used to estimate mortality. The competing risk model compared the lengths of hospital stays with the subdistribution hazard ratio (SHR).

**Results:** Overall, 938 patients were included in the analysis. The lactated Ringers group had a lower mortality rate (adjusted hazard ratio, 0.59; 95% CI 0.43-0.81) and shorter lengths of hospital stay (SHR, 1.39; 95% C.I. 1.15-1.67) than the saline group; the differences were greater in patients with chronic pulmonary disease and small and non-significant in those with chronic kidney disease, moderate to severe liver disease and cerebral vascular disease. The resuscitation efficacy was the same between fluid types, but serum lactate levels were significantly higher in the lactated Ringers group than in the saline group (0.12 mg/dl/h; 95% C.I.: 0.03, 0.21), especially in chronic liver disease patients. Compared to the saline group, the lactated Ringers group achieved target glucose level earlier in both diabetes and non-diabetes patients.

**Conclusion:** Lactate Ringer's solution provides greater benefits to patients with chronic pulmonary disease than to those with chronic kidney disease, or with moderate to severe liver disease. Comorbidities are important in choosing resuscitation fluid types.

## Introduction

The fourth edition of the Surviving Sepsis Campaign suggests using either balanced crystalloids or saline for fluid resuscitation in sepsis patients (1). Recently, a large, pragmatic randomized trial found that critically ill adult patients receiving balanced crystalloids had lower rates of the composite outcome of death and renal adverse events than those receiving saline (2). Our latest network meta-analysis also found a lower risk of mortality in sepsis patients treated with balanced crystalloids than in those treated with saline (3). In contrast, balanced crystalloids increased mortality in traumatic brain injury patients (3). Therefore, both the fluid types and patient condition need to be considered when choosing an optimal fluid treatment.

Saline and balanced crystalloids, such as lactated Ringers, have different fluid osmolalities (saline: isotonic, 308 mOsm/kg; lactated Ringers: hypotonic, 273 mOsm/kg) and contain different electrolyte composites and lactate metabolites, resulting in varying risks for ineffective increases in central venous pressure, hyperkalemia, acidosis or glucose instability, especially in sepsis patients with cormobidities (4). Saline solution contains no potassium or other chemical compounds, while lactated Ringers solution contains 4 mmol/L potassium and 28 mmol/L sodium lactate. The excessive potassium in lactated Ringers may specifically lead to hyperkalemia in chronic kidney disease patients (5). Seventy percent of serum lactate undergoes gluconeogenesis, which can potentially lead to an increase in blood glucose levels and instability, and these effects might be more significant in diabetes patients (6, 7). Thirty percent of serum lactate undergoes oxidation in the liver, producing ${\text{HCO}}_{3}^{-}$, which helps limit acidosis during sepsis. However, lactate metabolism is impaired in chronic liver disease patients, and thus, the acidosis prevention effect of lactated Ringers in chronic liver disease patients is questionable (8, 9).

This study aimed to investigate differences in mortality and lengths of stay in the intensive care unit (ICU) and hospital in sepsis patients treated with lactated Ringers or saline. Subgroup analyses of sepsis patients with different comorbidities, including chronic pulmonary disease, chronic kidney disease, chronic liver disease and diabetes, were also performed. The differences in trends of central venous pressure, central venous oxygen saturation, and the serum lactate level between fluid types were also investigated to compare their resuscitation efficacy. During and after the resuscitation periods, we compared the trends of serum potassium in patients with and without chronic kidney disease, the trends of blood glucose levels in diabetes and non-diabetes patients, and the trends of serum lactate in patients with and without chronic liver disease.

## Methods

### Study Population and Care Protocols

This study was conducted at medical ICU of a tertiary-care referral hospital with 1,514 beds in central Taiwan. For sepsis patients who met the criteria (10), we implemented bundled care and included them in this study. The sepsis bundles include (1) guided fluid resuscitation considering lactate clearance, mixed venous oxygen saturation, and urine volume; (2) early empiric antibiotic prescription rules, (3) glucose control protocols, (4) protective ventilator settings, (5) c-reactive protein, procalcitonin, B-type natriuretic peptide, HbA1C, and albumin levels before fluid resuscitation, and (6) lactate and mixed venous oxygenation levels, which are checked every 6 h for every sepsis patient in the first 24 h. Among the bundles, the glucose target was 140~180 mg/dl. An insulin sliding scale was applied for glucose levels higher than 180 mg/dl. The frequency of the one-touch glucose examination was every 4 h if the blood glucose level was lower than 180 mg/dl or every 2 h if the blood glucose level was higher than 180 mg/dl.

Databases of the sepsis management registry and electronic medical records which were collected prospectively were used for retrospective analysis. The protocol was approved by the Institutional Review Board of the Veteran General Hospital Taichung (IRB no. CF16017A). According to their resuscitation fluid profiles in the first 24 h, patients were divided into two groups: the saline group, who received mainly saline for resuscitation and <500 ml of lactated Ringers, and the lactated Ringers group, who received more than 500 ml of lactated Ringers in the first 24 h. In the sensitivity analysis, we further separated patients into three groups: the saline only, saline predominant (more saline in the total fluid amount) and lactated Ringers predominant groups (more lactated Ringers in the total fluid amount). Definitions of comorbidities, including chronic kidney disease, diabetes, mild or moderate to severe liver disease, congestive heart failure, and cerebral vascular disease, followed those of the Charlson Comorbidity Index (11) (Supplementary Table 1). We also recorded blood transfusion volumes, including red blood cells and fresh frozen plasma, and hemodialysis events for every patient.

### Statistical Methods for Mortality and Lengths of Stay in the ICU and Hospital

The differences in patient characteristics between two fluid type groups were compared by using Pearson's chi-squared test for categorical variables and Student's *t*-test for continuous variables. As our cohort was not randomly assigned to the two fluid type groups, we used propensity-score matching by fluid type to control for potential confounding factors and selection bias (12), thereby optimizing comparability between the saline group and lactated Ringers group (13). We entered the APACHE score, age and HbA1c level into the logistic regression analysis to compute propensity scores for each participant. The propensity score represented the probability of a patient with sepsis being assigned to the lactated Ringers group. Based on the propensity score, patients who received lactated Ringers were matched with two patients who received saline, and then we created propensity score-matched sets for the Cox proportional hazard model to estimate the hazard ratio of 90-day mortality. In addition, we also performed a logistic regression analysis to compare 90-day mortality between the saline group and lactated Ringers group (14). Finally, we used the competing risk model to estimate the subdistribution hazard ratios (SHRs) for the lengths of ICU and hospital stays (15, 16). We considered death a competing risk, and discharge from the ICU or hospital was the event of interest. We hypothesized that resuscitation fluid with fewer complications would lead to a shorter length of ICU stay and earlier discharge. We also compared the lengths of ICU stay and hospital stay between patients with and without comorbidities.

### Statistical Method for Repeated Measured Data

Mixed-effect linear models were used to analyze changes in the following clinical variables during and after the resuscitation period: central venous pressure, central venous oxygen saturation, serum bicarbonate, serum lactate, creatinine, urine output, serum potassium and blood glucose. Interaction terms between fluid types and changes in those clinical variables were included to investigate whether patients receiving these two fluids showed different trends. All statistical tests were performed with Stata version 14.0 (StataCorp, Texas, USA).

### Statistical Method for Glycemic Variability

We used the mean amplitude of glycemic excursions (MAGE) (17) and coefficient of variation (CoV) (18) to assess glycemic variability. The MAGE represents the mean blood glucose value exceeding the standard deviation from the 24-h mean blood glucose level, and CoV represents the ratio of the standard deviation to the mean glucose level. A high blood glucose index and low blood glucose index represent the risks of hyperglycemia and hypoglycemia, respectively (19). The glucose index was derived from a logarithmic transformation of the blood glucose scale that assigned maximum risk to blood glucose of 20 and 600 ml/dl and zero risk to 112.5 mg/dl (20). The above glycemic parameters were calculated using EasyGV software (21).

## Results

### Baseline Patient Characteristics

**Figure 1:**
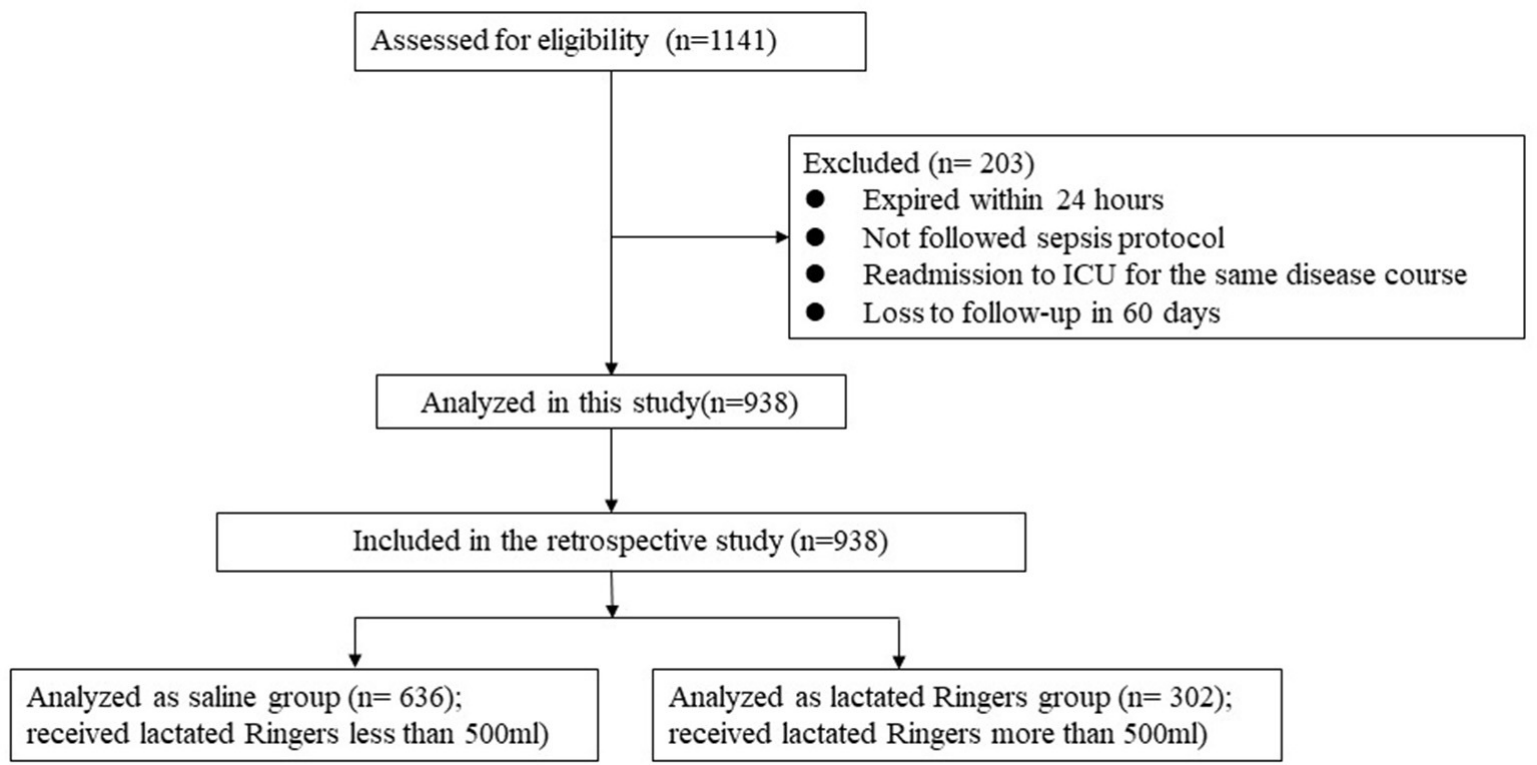
STrengthening the Reporting of OBservational studies in Epidemiology (STROBE) flow chart of study participants.

We assessed 1,141 cases for eligibility and included 938 sepsis patients for analysis (Figure 1). The saline group and lactated Ringers group included 636 and 302 patients, with male percentages of 76.1 and 73.8%, mean ages of 71.9 and 70.7, and mean Acute Physiology and Chronic Health Evaluation (APACHE) scores of 26.0 ± 6.9 and 29.0 ± 6.4, respectively (Table 1). The total fluid resuscitation volume on day 1 was not significantly different between the saline and lactated Ringers groups (4,591 vs. 4,959 ml, *p* = 0.157). The results of laboratory tests, including HbA1c, procalcitonin, c-reactive protein, B-type natriuretic peptide and albumin, before fluid resuscitation were not significantly different between the two groups (Table 1).

**Table 1:** Patient's characteristics according to types of fluid resuscitation.

	**Saline group**	**Lactated Ringer's group**	***P*-value**
No. of case	636	302	
**Gender**, ***n*** **(%)**			
Male	484 (76.1)	223 (73.8)	0.466
Female	152 (23.8)	79 (26.2)	
Age, mean (SD)	71.9 (15.8)	70.7 (15.3)	0.277
Body mass index, mean (SD)	23.2 (4.4)	23.0 (4.6)	0.613
APACHE II score, mean (SD)	26.0 (6.9)	29.0 (6.4)	<0.001
Total fluid resuscitation in day 1, mean (SD)	4,591 (3,778)	4,959 (3,602)	0.157
Saline fluid amount in day 1, mean (SD)	4,587 (3,776)	1,787 (2,362)	<0.001
Lactated Ringer's fluid amount in day 1, mean (SD)	3.9 (44.2)	3,172 (2442)	<0.001
**Comorbidity**			
Coronary artery disease, *n* (%)	68 (10.7)	26 (8.6)	0.353
Congestive heart failure, *n* (%)	156 (24.5)	68 (22.5)	0.513
Cerebral vascular disease, *n* (%)	214 (33.6)	83 (31.0)	0.413
Chronic pulmonary disease, *n* (%)	318 (50)	141 (46.7)	0.364
Chronic kidney disease, *n* (%)	45 (7.1)	33 (10.9)	0.057
Chronic liver disease, *n* (%)	29 (4.6)	15 (5.0)	0.869
Diabetes, *n* (%)	316 (49.7)	135 (44.7)	0.162
**Laboratory exam before resuscitation**			
HbA1C (%), mean (SD)	6.3 (1.4)	6.5 (1.5)	0.186
Procalcitonin (ng/ml), mean (SD)	18.7 (31.0)	18.8 (31.3)	0.979
c-reactive protein (mg/dl), mean (SD)	15.7 (11.7)	14.5 (11.3)	0.162
B-type natriuretic peptide (pg/ml), mean (SD)	8,911 (10,659)	8,385 (10,859)	0.562
Albumin (g/dl), mean (SD)	2.6 (0.7)	2.7 (0.6)	0.013

*SD, Standard deviation; APACHE, Acute Physiology and Chronic Health Evaluation*.

### Mortality and Lengths of ICU and Hospital Stays

**Figure 2:**
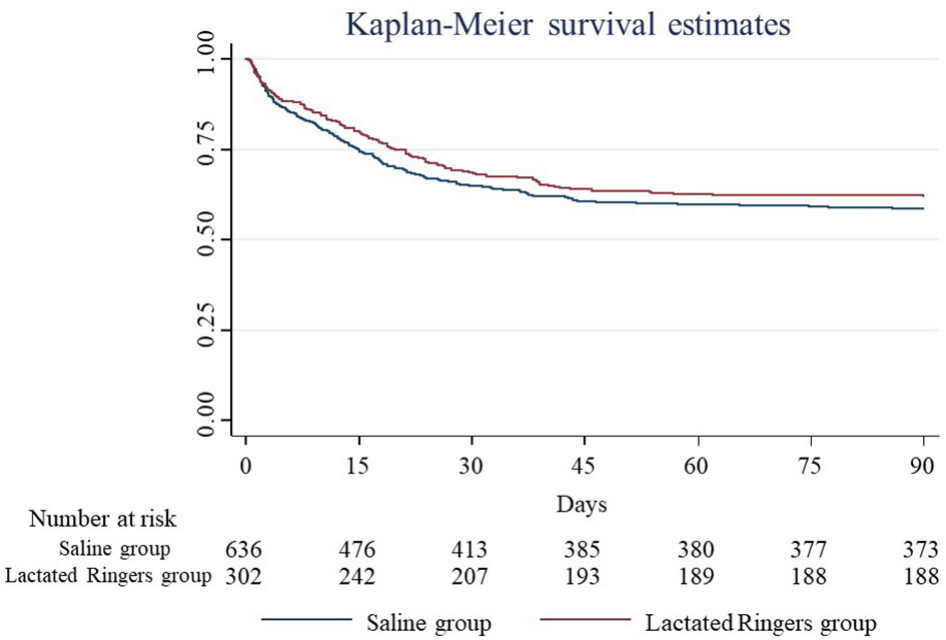
Kaplan-Maier curve for 90-day mortality.

Overall Kaplan-Maier curve for 90-day mortality between saline and lactated Ringers was shown in Figure 2. The propensity score distributions between the saline group and lactated Ringers group were similar after matching (Supplementary Table 2). In the propensity-score matched Cox regression analysis, the lactated Ringers group had a lower risk of mortality than the saline group (adjusted hazard ratio, 0.59; 95% CI 0.43-0.81). In the logistic regression analysis for 90-day mortality, the lactated Ringers group also had lower odds of mortality than the saline group (adjusted odds ratio, 0.68; 95% CI 0.51-0.92). In the competing risk regression analysis for length of ICU stay, patients resuscitated with lactated Ringers were discharged from the ICU (SHR, 1.41; 95% CI 1.17-1.71) and hospital (SHR, 1.39; 95% C.I. 1.15-1.67) earlier than those resuscitated with saline. We divided patients into three subgroups according to the saline fluid amount they received. The results showed that both the lactated Ringers predominant group (SHR, 1.49; 95% CI 1.18-1.88) and saline predominant group (SHR, 1.32; 95% CI 1.02-1.71) had significantly shorter ICU stays than the saline group (Table 2). Regarding the subgroup analyses stratified by comorbidities (Table 3), the use of lactated Ringers, compared to the use of saline, significantly reduced the length of stay in the ICU in patients with chronic pulmonary disease (SHR, 1.80; 95% CI 1.37, 2.36), without chronic kidney disease (SHR, 1.48; 95% CI 1.21, 1.80), without moderate to severe liver disease (SHR, 1.44; 95% CI 1.19, 1.75), and without cerebral vascular disease (SHR, 1.54; 95% CI 1.22, 1.95). No significant differences were found between the two types of fluids in patients without chronic pulmonary disease (SHR, 1.17; 95% CI 0.89, 1.53), with chronic kidney disease (SHR, 077; 95% CI 0.35, 1.70), with moderate to severe liver disease (SHR, 1.01; 95% CI 0.32, 3.17), and with cerebral vascular disease (SHR, 1.27; 95% CI 0.92, 1.76).

**Table 2:** Mortality analysis and competing risk analysis for length of stay in intensive care unit and hospital.

**Propensity score matching cox regression analysis for 90-days mortality**			
**Outcome**	**Comparison**	**Adjusted hazard ratio**	***P*** **-value**
90 days mortality	Lactated Ringer's group vs. saline group	0.59 (0.43-0.81)	<0.001
**Logistic regression analysis for 90-days mortality**			
**Outcome**	**Comparison**	**Adjusted odd ratio**	***P*** **-value**
90 days mortality	Lactated Ringer's group vs. saline group	0.68 (0.51, 0.92)	0.013
	APACHE	1.09 (1.07, 1.12)	<0.001
**Logistic regression analysis for 90-days mortality with three saline exposure level**			
**Outcome**	**Comparison**	**Adjusted odd ratio**	***P*** **-value**
90 days mortality	Saline predominant vs. saline only group	0.74 (0.50, 1.11)	0.142
	Lactated Ringer's predominant vs. saline only group	0.64 (0.44, 0.93)	0.019
	APACHE	1.09 (1.07, 1.12)	<0.001
**Competing risk analysis for ICU and hospital stay**			
**Outcome**	**Comparison**	**SHR (95% C.I.)**	***P*** **-value**
Length of ICU day	Lactated Ringer's group vs. saline group	1.41 (1.17, 1.71)	<0.001
	15.9 (13.7~16.1) vs. 17.8 (16.6~19.7)		
	APACHE	0.95 (0.93, 0.96)	<0.001
Length of hospital day	Lactated Ringer's group vs. saline group	1.39 (1.15, 1.67)	<0.001
	26.0 (23.2~28.0) vs. 29.7 (27.9~32.2)		
	APACHE	0.95 (0.93, 0.96)	<0.001
**Competing risk analysis for ICU and hospital stay with three saline exposure level**			
**Outcome**	**Comparison**	**SHR (95% C.I.)**	***P*** **-value**
Length of ICU day	Lactated Ringer's predominant vs. saline only group	1.49 (1.18, 1.88)	0.001
	15.3 (13.4~16.3) vs. 17.8 (16.6~19.7)		
	Saline predominant vs saline only group	1.32 (1.02, 1.71)	0.034
	15.0 (12.5~16.9) vs. 17.8 (16.6~19.7)		
	APACHE	0.95 (0.93, 0.96)	<0.001
Length of hospital day	Lactated Ringer's predominant vs. saline only group	1.44 (1.14, 1.81)	0.002
	24.5 (22.0~28.0) vs. 29.7 (27.9~32.2)		
	Saline predominant vs. saline only group	1.32 (1.01, 1.71)	0.037
	27.0 (23.5~29.7 vs. 29.7 (27.9~32.2)		
	APACHE	0.95 (0.93, 0.96)	<0.001

*SHR, subdistribution hazard ratio*.

**Table 3:** Competing risk analysis for length of stay in intensive care unit and hospital in different comorbidities.

**Comorbidities**	**No. of cases**	**Length of ICU day**		**Length of hospital day**	
		SHR^*^ (95% C.I.)	***P*-value**	SHR^*^ (95% C.I.)	***P*-value**
**Chronic pulmonary disease (CPD)**					
No CPD	467	1.17 (0.89, 1.53)	0.258	1.13 (0.87, 1.48)	0.356
With CPD	447	1.80 (1.37, 2.36)	<0.001	1.78 (1.36, 2.34)	<0.001
**Chronic kidney disease (CKD)**					
No CKD	838	1.48 (1.21, 1.80)	<0.001	1.44 (1.18, 1.75)	<0.001
With CKD	76	0.77 (0.35, 1.70)	0.525	0.82 (0.36, 1.85)	0.635
**Acute kidney injury or CKD**					
No AKI or CKD	373	1.35 (1.04, 1.76)	0.026	1.34 (1.02, 1.75)	0.032
With AKI or CKD	528	1.28 (0.96, 1.70)	0.094	1.25 (0.94, 1.66)	0.125
**Mild liver disease (LD)**					
No liver disease	676	1.38 (1.11, 1.72)	0.004	1.36 (1.09, 1.69)	0.006
With mild liver disease	238	1.49 (1.02, 2.18)	0.039	1.42 (0.97, 2.06)	0.068
**Moderate to severe LD**					
No liver disease	873	1.44 (1.19, 1.75)	<0.001	1.41 (1.16, 1.71)	<0.001
With moderate to severe LD	41	1.01 (0.32, 3.17)	0.984	1.01 (0.33, 3.40)	0.924
**Cerebral vascular disease (CVD)**					
No CVD	615	1.54 (1.22, 1.95)	<0.001	1.52 (1.20, 1.93)	0.001
With CVD	299	1.27 (0.92, 1.76)	0.148	1.24 (0.90, 1.71)	0.183
**Congestive heart failure (CHF)**					
No CHF	696	1.39 (1.11, 1.72)	0.003	1.36 (1.09, 1.68)	0.006
With CHF	218	1.54 (1.05, 2.27)	0.028	1.55 (1.05, 2.28)	0.026
**Diabetes mellitus**					
No diabetes mellitus	472	1.41 (1.09, 1.81)	0.008	1.43 (1.10, 1.84)	0.007
With diabetes mellitus	442	1.43 (1.07, 1.91)	0.015	1.34 (1.01, 1.77)	0.041
**Rheumatology disease (RD)**					
No RD	838	1.32 (1.08, 1.61)	0.006	1.30 (1.07, 1.59)	0.009
With RD	76	2.71 (1.34, 5.51)	0.006	2.35 (1.26, 4.38)	0.007
**Malignancy**					
No Malignancy	505	1.47 (1.16, 1.87)	0.002	1.46 (1.14, 1.85)	0.002
With Malignancy	409	1.33 (0.98, 1.80)	0.071	1.27 (0.94, 1.71)	0.122

* *SHR, Subdistribution hazard ratio; lactated Ringer's group compared to saline group (reference group)*.

### Relationships Between Fluid Types and Clinical Variables Associated With Resuscitation

**Figure 3:**
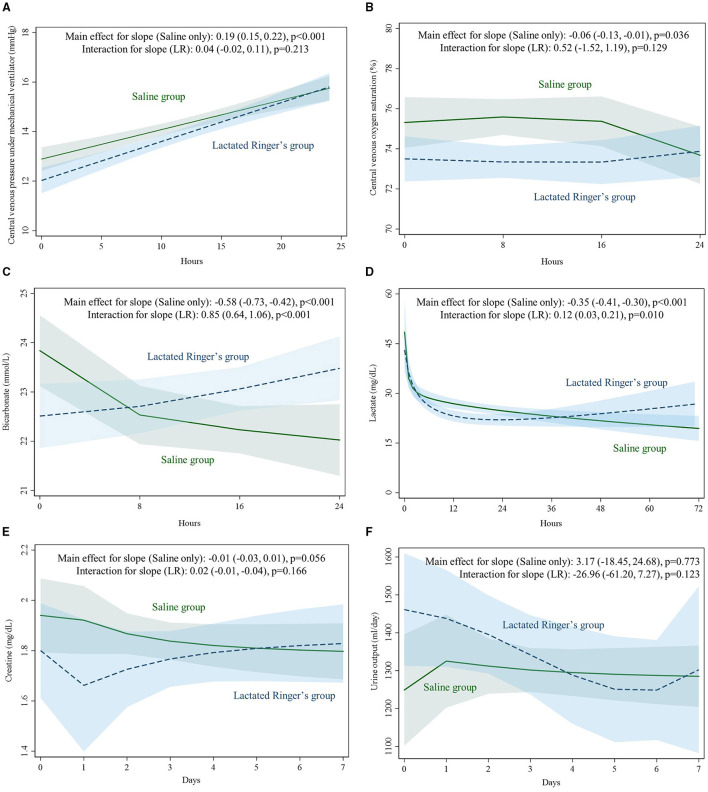
**(A–F)** Trends for central venous pressure, central venous oxygen saturation, serum bicarbonate, lactate, creatinine changes and urine output among fluid types and diabetes status. LR, lactated Ringer's.

Central venous pressure trends consistently increased in the first 24 h (0.19 mmHg/h; 95% C.I.: 0.15, 0.55) in both the saline group and lactated Ringers group, and the trends were not significantly different between the two groups (0.04 mmHg/h; 95% C.I.: −0.02, 0.11) (Figure 3A). Central venous oxygen saturation trends were also not significantly different between the two groups (0.52%/h, 95% C.I.: −1.2, 1.19) (Figure 3B). Serum bicarbonate progressively decreased in the saline group (−0.58 mmol/L/h; 95% C.I.: −0.73, −0.42) but increased significantly in the lactated Ringers group (0.85 mmol/L/h; 95% C.I.: 0.64, 1.06) (Figure 3C). The serum lactate levels decreased in the first few hours in both groups; it continued to decrease for 72 h in the saline group (−0.35 mg/dl/h; 95% C.I.: −0.41, −0.30) but remained abnormally high in the lactated Ringers group from 12 to 72 h (0.12 mg/dl/h; 95% C.I.: 0.03, 0.21) (Figure 3D). For the first 7 days, serum creatinine and urine output trends were not significantly different between the saline group and lactated Ringers group (Figures 3E,F).

### Relationships Between Fluid Types and Serum Potassium and Blood Glucose

**Figure 4:**
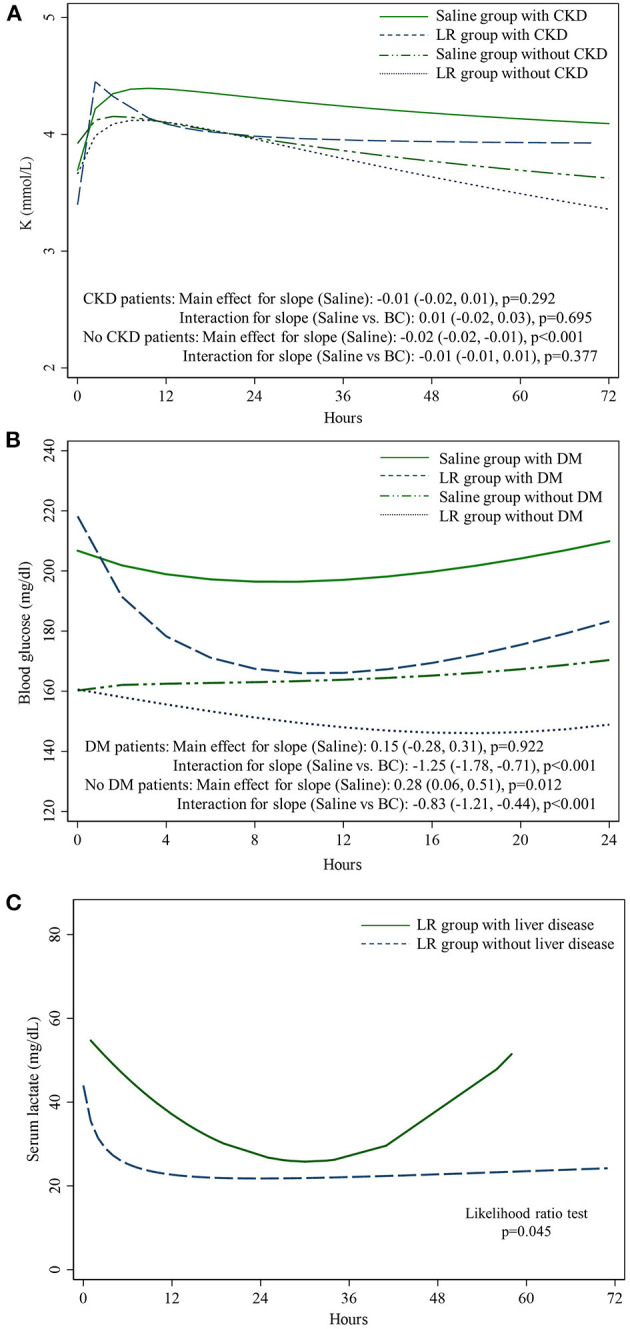
Trends for **(A)** serum potassium among fluid types and chronic kidney status, **(B)** blood sugar changes among fluid types and diabetes status, **(C)** serum lactate in patient with or without moderate to severe liver disease. K, potassium; LR, lactated Ringer's; CKD, chronic kidney disease; DM, diabetes mellitus.

Serum potassium levels were higher than normal on admission to the ICU and gradually decreased in the first 72 h in the saline subgroup without chronic kidney disease (−0.02 mmol/L/h; 95% CI: −0.02, −0.01) but remained in the normal upper limit in the saline subgroup with chronic kidney disease (−0.01 mmol/L/h; 95% CI: −0.02, 0.01). The serum potassium trends between the saline group and lactated Ringers group were not significantly different in patients with or without chronic kidney disease (Figure 4A). Blood glucose in the first 24 h consistently remained higher than normal in the saline group in both diabetes (0.15 mg/dl/h; 95% CI: −0.28, 0.31) and non-diabetes patients (0.28; 95% CI: 0.06, 0.51); in the lactated Ringers group, the blood glucose level approached the target level in both patients with diabetes (−1.25 mg/dl/h; 95% CI: −1.78, −0.71) and patients without diabetes (−0.83 mg/dl/h; 95% CI: −1.21, −0.44) (Figure 4B). In patients receiving lactated Ringers, the serum lactate level was significantly higher in patients with moderate to severe liver disease than in patients without liver disease (*p* = 0.045) (Figure 4C).

### Relationships Between Fluid Types and Glycemic Variability (MAGE, CoV)

Compared to the saline group, the lactated Ringers group had a significantly lower glucose CoV (−1.78%, 95% CI: −3.34, −0.23) and a lower MAGE (−0.51%, 95% CI: −0.89, −0.13). The high blood glucose index was significantly lower in the lactated Ringers group than in the saline group (−3.17, 95% CI: −4.40, −1.95), but the low blood glucose index was not significantly different (0.03, 95% CI: −0.66, 0.71) (Table 4).

**Table 4:** Adjusted glycemic variability and fluid types.

	**Diabetes mellitus**	**APACHE**	**Lactated Ringer's group vs. Saline only**
CoV (%)	1.64 (0.21, 3.08)^*^	0.14 (0.03, 0.24)^**^	−1.78 (−3.34, −0.23)^*^
MAGE	0.41 (0.06, 0.75)^*^	0.02 (−0.01, 0.05)	−0.51 (−0.89, −0.13)^**^
HBGI	5.47 (4.35, 6.60)^***^	0.05 (−0.04, 0.13)	−3.17 (−4.40, −1.95)^***^
LBGI	−0.22 (−0.85, 0.41)	0.09 (0.04, 0.13)^***^	0.03 (−0.66, 0.71)

* *<0.05*,

** *<0.01*,

*** *<0.001*.

*CoV, coefficient of variation; MAGE, mean amplitude of glycemic excursion; HBGI, High Blood Glucose Index; LBGI, Low Blood Glucose Index*.

The proportions of patients requiring hemodialysis in the first 7 days were 27.4% in the saline group and 28.5% in the lactated Ringers group. The red blood cell transfusion volumes in the first 7 days were 443.4 ml (95% CI: 405.79, 480.99) in the saline group and 427.98 ml (95% CI: 370.75, 485.21) in the lactated Ringers group. The fresh frozen plasma transfusion volume in the first 7 days was significantly higher in the saline group (0.55 units, 95% CI: 0.45, 0.64) than in the lactated Ringers group (0.36 units, 95% CI: 0.22, 0.50) (Supplementary Table 3).

## Discussion

This prospective cohort study found that using lactated Ringers solution for resuscitation in sepsis patients decreased mortality and shortened the lengths of ICU and hospital stays compared with using saline, especially in patients with chronic pulmonary disease, without chronic kidney disease, without moderate to severe liver disease and without cerebral vascular disease. The trends for central venous pressure and oxygen saturation during the resuscitation period were similar between the saline group and lactated Ringers group, but the serum lactate level after resuscitation was significantly higher in the lactated Ringers group, especially in the chronic liver disease subgroup. The serum potassium level increased in the first few hours but recovered more slowly in patients with chronic kidney disease regardless of fluid type. Blood glucose reached the target level faster and the glycemic variability was lower in the lactated Ringers group, which may suggest that lactated Ringers helps stabilize blood glucose levels during and after resuscitation.

Our analysis further confirmed that sepsis patients treated with saline had increased mortality compared to those treated with lactated Ringers (2). Evidence has also suggested that saline prolongs the ICU stay and hospital stay due to the increased risk of hyperchloremia acidosis, endothelial glycocalyx damage-related interstitial edema, renal vessel constriction-related kidney injury, and the need for blood product transfusion (22). However, the reduced risk of acidosis and shortened length of ICU stay associated with the use of lactated Ringers were observed in only sepsis patients without chronic kidney disease and without chronic liver disease. This implies that intact kidney and liver functions play important roles in the efficacy of lactated Ringers in inducing acidosis prevention effects. On the other hand, the use of lactated Ringers shortened the ICU length of stay in sepsis patients with chronic pulmonary disease but not in those without; this phenomenon seems to suggest that the acidosis prevention effect of lactated Ringers is especially important for patients with chronic pulmonary disease who often and easily develop respiratory acidosis.

The difference in osmolality did not seem to affect resuscitation efficacy, as the trends of central venous pressure and central venous oxygen saturation were not significantly different between the two fluid groups. However, the lactate levels during and after resuscitation were higher in the lactated Ringers group. This indicates that lactated Ringers may increase the serum lactate level during the resuscitation period. Thus, we cannot use the decreasing trend of serum lactate as a surrogate for perfusion restoration and resuscitation efficacy, especially in patients who receive lactated Ringers as the main resuscitation fluid (23).

The risk of hyperkalemia in patients who receive lactated Ringers, especially those with chronic kidney disease, is a concern. Our analysis found that the serum potassium level increased in the first few hours, and the time to return to normal was not different between the saline and lactated Ringers groups. However, the rate of recovery was slower in chronic kidney disease patients. Another study found that the hyperkalemia risk was lower in chronic kidney disease patients among patients who received lactated Ringers (24). This implies that the acid-base effects of saline are more important for serum potassium homeostasis than those induced by the small amount of potassium in lactated Ringers fluid.

Lactated Ringers solution delays glycemic recovery in diabetic ketoacidosis, but acidosis recovery is faster with the administration of lactated Ringers solution (7). Our study found that in the lactated Ringers group, the initially high glucose level decreased to the target glucose level faster than that in the saline group. Lactated Ringers also decreased glycemic variability and decreased the risk for high blood glucose. This could be explained by the fact that the balanced electrolyte distribution in lactated Ringers may help stabilize blood glucose levels. Finally, in patients with moderate to severe liver disease, lactate metabolism and thus the acidosis-prevention effect were impaired. This may explain why the lactate level remained high during and after resuscitation in liver disease patients.

The strength of this study was that we collected detailed comorbidity data and implemented standard care bundles, including regularly evaluating resuscitation targets to adjust fluid volume administration, collecting baseline laboratory data, and implementing standard glucose control and glucose target protocols. This helped to control all possible confounding factors. Our study also had some limitations. First, the choice of lactated Ringers or saline was dependent on physician preference. Second, resuscitation fluid volume and types before ICU admission were not available. The APACHE score in the lactated Ringers group was higher than that in the saline group. We consider this confounding factor in the adjusted regression model.

## Conclusions

Lactated Ringers use was associated with lower mortality and shorter lengths of ICU and hospital stays, and these beneficial effects were observed in patients with chronic pulmonary disease, without chronic kidney disease, without chronic liver disease and without cerebral vascular disease. Lactated Ringers use was associated with higher serum lactate levels, especially in liver disease patients. The glucose levels during resuscitation were highest in diabetes patients receiving saline and lowest in non-diabetes patients receiving lactated Ringers.

## Data Availability Statement

The original contributions presented in the study are included in the article/Supplementary Material, further inquiries can be directed to the corresponding author.

## Ethics Statement

The protocol was approved by the Institutional Review Board of the Veteran General Hospital Taichung (IRB no. CF16017A). Written informed consent was not provided because identifications were deidentified by hospital.

## Author Contributions

C-HT: data curation and writing—original draft preparation. T-TC: conceptualization, methodology, data curation, and conceptualization. M-CC: conceptualization and validation. K-YC and S-MW: data curation and conceptualization. M-CS: software and validation. Y-KT: supervision conceptualization, reviewing, and editing. All authors contributed to the article and approved the submitted version.

## Funding

This work was partly funded by a grant from the Ministry of Science and Technology of Taiwan (grant no. MOST 106-2314-B-002-098-MY3).

## Conflict of Interest

The authors declare that the research was conducted in the absence of any commercial or financial relationships that could be construed as a potential conflict of interest.

## Publisher's Note

All claims expressed in this article are solely those of the authors and do not necessarily represent those of their affiliated organizations, or those of the publisher, the editors and the reviewers. Any product that may be evaluated in this article, or claim that may be made by its manufacturer, is not guaranteed or endorsed by the publisher.

## Abbreviations

SHR: subdistribution hazard ratio
ICU: intensive care unit
APACHE: Acute Physiology and Chronic Health Evaluation
HbA1c: Hemoglobin A1c
CoV: coefficient of variation
MAGE: mean amplitude of glycemic excursion.
