# Supplementary material for: Impact of Comorbidities on Beneficial Effect of Lactated Ringers vs. Saline in Sepsis Patients
Source: Front Med (Lausanne). 2021 Dec 13;8:758902. doi: 10.3389/fmed.2021.758902 (PMC8710469; doi:10.3389/fmed.2021.758902)
Supplement: Supplementary file 1 [file Data_Sheet_1.docx]

Supplementary Table 1. Definitions for comorbidities

| Comorbidities | Definition |
| --- | --- |
| Chronic kidney disease | IC9-CM diagnosis codes: 403.01, 403.11, 403.91, 404.02, 404.03, 404.12, 404.13, 404.92, 404.93  583.0, 583.1, 583.2, 583.4, 583.5, 583.6, 583.7, 588.0, V42.0, V45.1 |
| Acute kidney injury or Chronic kidney disease | Serum creatinine >1.5mg/dl or IC9-CM diagnosis codes:  403.01, 403.11, 403.91, 404.02, 404.03, 404.12, 404.13, 404.92, 404.93  583.0, 583.1, 583.2, 583.4, 583.5, 583.6, 583.7, 588.0, V42.0, V45.1 |
| Diabetes | HbA1C>6.5 during admission or IC9-CM diagnosis codes: 250.0 - 250.9 |
| Mild liver disease | IC9-CM diagnosis codes: 070.22, 070.23, 070.32, 070.33, 070.44, 070.54, 070.6, 070.9, 573.3, 573.4, 573.8, 573.9, V42.7, 570.X, 571.X |
| Moderate or severe liver disease | IC9-CM diagnosis codes: 456.0, 456.1, 456.2, 572.2, 572.3, 572.4, 572.5, 572.6, 572.7, 572.8 |
| Congestive heart failure | IC9-CM diagnosis codes: 398.91, 402.01, 402.11, 402.91, 404.01, 404.03, 404.11, 404.13, 404.91, 404.93, 425.4, 425.5, 425.6, 425.7, 425.8, 425.9, 428.X |
| Cerebral vascular disease | IC9-CM diagnosis codes: 430.X, 431.X, 432.X, 433.X, 434.X, 435.X, 436.X, 437.X, 438.X, 362.34 |
| Chronic pulmonary disease | IC9-CM diagnosis codes: 416.8, 416.9, 506.4, 508.1, 508.8, 490.X, 491.X, 492.X, 493.X, 494.X, 495.X, 496.X, 497.X, 498.X, 499.X, 500.X, 501.X, 502.X, 503.X, 504.X, 505.X |
| Rheumatology disease | IC9-CM diagnosis codes: 446.5, 710.0, 710.1, 710.2, 710.3, 710.4, 714.0, 714.1, 714.2, 714.8 |
| Malignancy | IC9-CM diagnosis codes: 140.X, 141.X, 142.X, 143.X, 144.X, 145.X, 146.X, 147.X, 148.X, 149.X, 150.X, 151.X, 152.X, 153.X, 154.X, 155.X, 156.X, 157.X, 158.X, 159.X, 160.X, 161.X, 162.X, 163.X, 164.X, 165.X, 170.X, 171.X, 172.X, 174.X, 175.X, 176.X, 179.X, 180.X, 181.X, 182.X, 183.X, 184.X, 185.X, 186.X, 187.X, 188.X, 189.X, 190.X, 191.X, 192.X, 193.X, 194.X, 200.X, 201.X, 202.X, 203.X, 204.X, 205.X, 206.X, 207.X, 208.X, 195.1, 195.2, 195.3, 195.4, 195.5, 195.6, 195.7, 195.8, 238.6 |

Supplementary table 2. Kernel Density Plot of Propensity Score


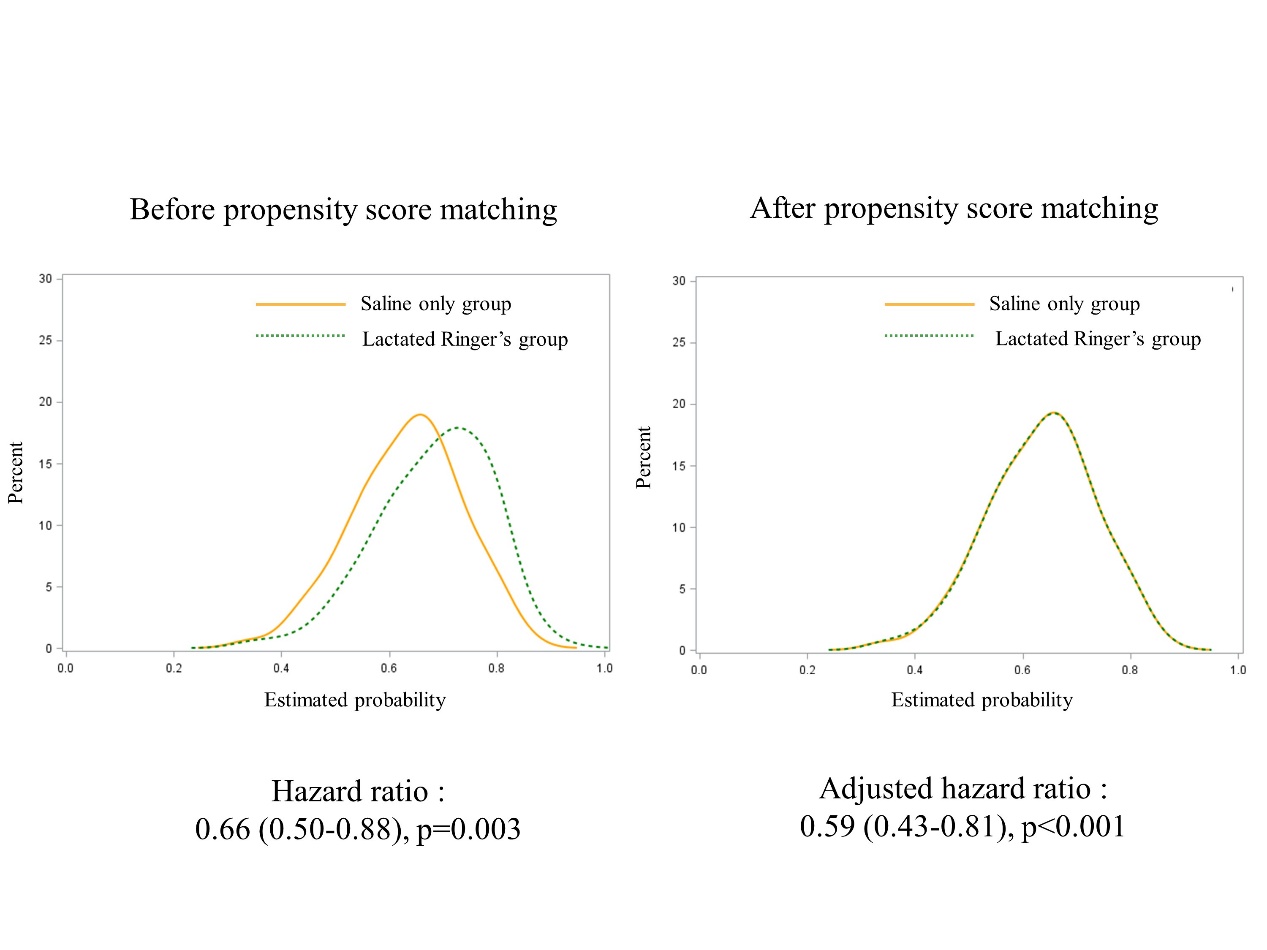


*Parameters for propensity score matching: age, HbA1C, APACHE, baseline sugar level

Supplementary Table 3. Blood transfusion and fluid types

|  | Saline only group | Lactated Ringer’s group | P value |
| --- | --- | --- | --- |
| Day 0-7 Hemodialyasis | 174 (27.4%) | 86 (28.5%) | 0.755 |
| Day 0-7 Red blood cell  transfusion volume | 443.40 ml  (405.79~480.99) | 427.98  (370.75~485.21) | 0.653 |
| Day 0-7 Fresh frozen plasma  transfusion volume | 0.55 unit  (0.45~0.64) | 0.36 unit  (0.22~0.50) | 0.027 |
